# Confirmation of Dissipative Sensing Enhancement in a Microresonator Using Multimode Input †

**DOI:** 10.3390/s23218700

**Published:** 2023-10-25

**Authors:** Sreekul Raj Rajagopal, Limu Ke, Karleyda Sandoval, Albert T. Rosenberger

**Affiliations:** Department of Physics, Oklahoma State University, Stillwater, OK 74078, USA

**Keywords:** microresonator, whispering-gallery modes, dissipative sensing, multimode fiber

## Abstract

Optical microresonators have proven to be especially useful for sensing applications. In most cases, the sensing mechanism is dispersive, where the resonance frequency of a mode shifts in response to a change in the ambient index of refraction. It is also possible to conduct dissipative sensing, in which absorption by an analyte causes measurable changes in the mode linewidth and in the throughput dip depth. If the mode is overcoupled, the dip depth response can be more sensitive than the linewidth response, but overcoupling is not always easy to achieve. We have recently shown theoretically that using multimode input to the microresonator can enhance the dip-depth sensitivity by a factor of several thousand relative to that of single-mode input and by a factor of nearly 100 compared to the linewidth sensitivity. Here, we experimentally confirm these enhancements using an absorbing dye dissolved in methanol inside a hollow bottle resonator. We review the theory, describe the setup and procedure, detail the fabrication and characterization of an asymmetrically tapered fiber to produce multimode input, and present sensing enhancement results that agree with all the predictions of the theory.

## 1. Introduction

Over the past few decades, optical microresonators have been a topic of much research and application. They can have ultra-high quality factors (*Q*) and small mode volumes and hence have emerged as an extraordinary platform for various sensing applications [1,2]. In particular, owing to their high sensitivities and low detection limits, optical sensors based on whispering-gallery mode (WGM) microresonators have been used to monitor changes in various physical quantities such as pressure, temperature, chemical composition, and refractive index, as well as other quantities [3,4]. Optical WGM sensors operate by detecting changes in features of their throughput spectral response such as the resonance frequency, linewidth, and resonant throughput dip depth, due to perturbations in the environment probed by the evanescent (or interacting) fraction of the WGM [5]. The sensing principle of an optical WGM sensor can be broadly classified as dispersive or dissipative. The more widely used modality, dispersive sensing, is based on measuring the shift in the WGM resonance frequency due to a change in the ambient refractive index. Dissipative sensing, in general, entails measuring the change in the linewidth of a WGM due to absorption or scattering by an analyte in the ambient medium. However, dissipative phenomena can also induce a change in the resonant throughput dip depth, and hence, dissipative sensing can be realized by monitoring the change in the throughput dip depth of a WGM.

A tapered optical fiber enables the efficient coupling of light into and out of the microresonator. Usually, an adiabatically tapered fiber (symmetric tapering) is used, and hence, a single fiber mode is incident on the microresonator. The resonant throughput dip depth in such a system depends on the ratio of the outcoupling loss (*T*) to the effective intrinsic loss (*αL*). For typical systems used at a wavelength of 1550 nm, with a taper waist radius of 2 μm and a resonator radius of 300 μm, outcoupling into higher-order waist modes is negligible [6], and the tapered fiber acts as if it were a single-mode fiber. In earlier work, dissipative sensing was studied in detail in an adiabatically tapered-fiber-coupled microresonator system, and it was shown that, when overcoupled (*T* > *αL*), dissipative sensing based on the relative change in dip depth can be more sensitive than either dispersive sensing based on frequency change or linewidth-change-based dissipative sensing [7,8]. However, it is not always possible to achieve overcoupling, in which case there is no inherent sensitivity advantage to using the dip-depth sensing method. Recent studies on dissipative sensing based on the transmittance dip for two different configurations, a waveguide side coupled to a circular microresonator [9] and a self-interference microring resonator [10,11,12,13,14], have again confirmed that dissipative sensing can be more sensitive than dispersive sensing. However, in all the dissipative sensing configurations discussed to date, the input to the microresonator consists of a single waveguide mode or fiber mode.

We have recently shown theoretically that dissipative sensing in a microresonator can be enhanced by using two-mode input [15]. With two-mode input, dip-depth-based sensing can be two orders of magnitude more sensitive than linewidth-based sensing, and two-mode versus one-mode dip-depth sensing can be enhanced by three orders of magnitude. In the current paper, we experimentally confirm these results using an absorbing dye dissolved in methanol inside a hollow bottle resonator. The Section 2 presents a summary of the theory, a description of the experimental setup and procedure, and the specifics of achieving multimode input to the microresonator by using asymmetric tapering of the coupling fiber (nonadiabatic downtaper and adiabatic uptaper) to couple light into and out of the microresonator. It details the fabrication and modeling of, and beat length measurements made on, the asymmetric tapered fiber, along with delineation-curve arguments for its nonadiabaticity. The Section 3 presents experimental results and analysis, followed by a Section 4. The two enhancement factors that are measured in this work and compared to theory are *η*_21_, the two-mode dip-depth sensing response relative to the one-mode dip-depth sensing response, and *η_dl_*, the two-mode dip-depth sensing response relative to the linewidth sensing response. These enhancements are independent of *Q*, but absolute sensitivity increases with *Q*, so we also compare our system to an *ideal* one-mode system and show that a mode with an intrinsic quality factor *Q_i_* at least two orders of magnitude larger (~10^9^) would be needed to provide the same sensitivity, whether the single-mode system uses dip-depth or linewidth sensing.

## 2. Materials and Methods

### 2.1. Summary of Theory

As described in more detail in our theoretical treatment [15], the fiber that couples light into and out of the microresonator is tapered to a thin waist upon which two modes are excited owing to the nonadiabatic downtaper transition. The incoupling strength and outcoupling loss are assumed to be equal (we showed in [15] that this assumption is not restrictive) and given by *T*_1_ for the fundamental fiber mode and by *T*_2_ for the higher-order fiber mode. The two fiber modes have the same frequency and couple to a single microresonator WGM that has intrinsic loss *αL*, but they have different propagation constants and different amplitudes. The ratio of amplitudes, higher-order to fundamental, is denoted by *m*. When the frequency is tuned to WGM resonance, the throughput power depends on the relative phase of the two input modes, which can be selected by moving the microresonator along the fiber waist. The throughput power relative to the input power is given, on resonance, by

(1) $$R_{\pm }=\frac{{\left(T_{1}-T_{2}-\alpha L\pm 2\sqrt{T_{1}T_{2}}m\right)}^{2}}{{\left(T_{1}+T_{2}+\alpha L\;\right)}^{2}},$$

where *R*_+_ and *R*_−_ denote the relative throughput with the two fiber modes in phase and out of phase, respectively. These throughput powers relative to the input power in the fundamental fiber mode are measured by comparing to the value far off resonance, where no light couples into the WGM; the light in both fiber modes then encounters the adiabatic uptaper in which light cannot couple between modes, so only the light in the fundamental mode gets transmitted into the single-mode untapered fiber, and thus, *R* = 1 [15]. Under the conditions that lead to large sensing enhancement, a shallow dip is observed for in-phase coupling (*R*_+_ slightly less than 1), and a small peak occurs with out-of-phase coupling (*R*_−_ slightly greater than 1). This means that

(2) $$T_{1}\approx \sqrt{T_{1}T_{2}}m\approx T_{2}+\alpha L,$$

with values of the three parameters increasing from left to right. Sensing is carried out with in-phase coupling, where the relative dip depth is given by $M=1-R_{+}$. As a function of the intrinsic loss, the dip depth is

(3) $$M\left(\alpha L\right)=\frac{4\left[\left(T_{1}+\sqrt{T_{1}T_{2}}m\right)\left(T_{2}+\alpha L-\sqrt{T_{1}T_{2}}m\right)\right]\;}{{\left(T_{1}+T_{2}+\alpha L\;\right)}^{2}}.$$

The introduction of an absorbing analyte changes the effective intrinsic loss, resulting in a fractional change in the dip depth. For our case of a hollow bottle resonator [16,17] filled with methanol, the effective intrinsic loss is

(4) $$\alpha L=\alpha_{i}L+f\alpha_{s}L,$$

which, upon introducing the analyte, becomes

(5) $$\alpha^{\prime }L=\alpha_{i}L+f\alpha_{s}L+f\alpha_{a}L=\alpha L+d\alpha L,$$

where *α_i_* is the intrinsic loss coefficient of the microresonator that includes scattering, absorption, and radiation losses, *α_s_* is the absorption coefficient of the solvent that contains the analyte in a typical sensing experiment, and *α_a_* is the absorption coefficient of the analyte. The microresonator circumference is *L*, and *f* is the internal evanescent (interacting) fraction [5] of the WGM that interacts with the solvent and analyte. The change in effective internal loss is due to the addition of the analyte: *dαL* = *fα_a_L*.

When the fractional change in the throughput, |Δ*R*_+_/*R*_+_|, is small, the change in *M* will be linear in *dαL*, and the fractional change in the dip depth is positive (the dip gets deeper with an increasing analyte concentration) and can be written as

(6) $$\frac{1}{M}\frac{dM}{d\alpha L}=\frac{\left(T_{1}-T_{2}-\alpha L+2\sqrt{T_{1}T_{2}}m\right)}{\left(T_{1}+T_{2}+\alpha L\;\right)\left(T_{2}+\alpha L-\sqrt{T_{1}T_{2}}m\right)}.$$

This will be the case for two-mode input, where *R*_+_ remains near 1. Note, however, that for one-mode input using an adiabatically tapered fiber with the same waist radius, setting *m* = 0 in Equation (1) results in R≪1. For small enough analyte concentrations (as will be seen in the Section 3), the change in *M* will also be linear in the one-mode case, and thus, the sensitivity enhancement will be given by the ratio of Equation (6) with arbitrary *m* to Equation (6) with *m* = 0:

(7) $$\eta_{21}=\left|\frac{\left(T_{2}+\alpha L\right)\left(T_{1}-T_{2}-\alpha L+2\sqrt{T_{1}T_{2}}m\right)}{\left(T_{1}-T_{2}-\alpha L\;\right)\left(T_{2}+\alpha L-\sqrt{T_{1}T_{2}}m\right)}\right|,$$

where *η*_21_ is the sensitivity enhancement factor for two-mode input relative to one-mode input. Given the conditions of Equation (2), this enhancement can be quite large (>1000). The enhancement factor of Equation (7) can be written in more compact form in terms of the values of *R*_+_ and *R*_−_:

(8) $$\eta_{21}=\left|\frac{1-\frac{1}{2}\left(\sqrt{R_{+}}-\sqrt{R_{-}}\right)}{\sqrt{R_{+}}-\sqrt{R_{-}}}\right|\frac{2\sqrt{R_{+}}}{1-\sqrt{R_{+}}}.$$

If |Δ*R*_+_/*R*_+_| is not small, or if we wish to check the linearity of the dip-depth response, we insert Equation (5) into Equation (3) and replace the *dM*/*M* found as in Equation (6) for *m* ≠ 0 with

(9) $$\frac{\Delta M}{M}=\frac{M(\alpha^{\prime }L)-M(\alpha L)}{M(\alpha L)}.$$

Then, *η*_21_ is equal to the ratio of Equation (9) to the *dM*/*M* of Equation (6) with *m* = 0.

To compare the two-mode dip-depth sensing response to the linewidth sensing response, which is the same in the two-mode and one-mode cases, begin with the relationship between the linewidth and the total loss:

(10) $$\Delta \nu =\frac{c}{4\pi^{2}na}\left(T_{1}+T_{2}+\alpha L\;\right),$$

where *c* is the speed of light, *n* is the effective refractive index of the WGM, and *a* is the microresonator radius. From this, it is clear that

(11) $$\frac{1}{\Delta \nu }\frac{d\Delta \nu }{d\alpha L}=\frac{1}{T_{1}+T_{2}+\alpha L},$$

and so the enhancement of dip-depth sensing relative to linewidth sensing in the two-mode case is given by the ratio of Equation (6) to Equation (11):

(12) $$\eta_{dl}=\frac{T_{1}-T_{2}-\alpha L+2\sqrt{T_{1}T_{2}}m}{T_{2}+\alpha L-\sqrt{T_{1}T_{2}}m}=\frac{2\sqrt{R_{+}}}{1-\sqrt{R_{+}}},$$

which can be on the order of 100.

Finally, we note that neither enhancement factor, *η*_21_ or *η_dl_*, depends on the quality factor *Q*. However, the absolute sensitivity will increase as *Q* does, as is evident from the inverse dependence on the total loss in Equations (6) and (11). In [15], we showed that in the *ideal* one-mode case (*m* = 0, *T*_2_ = 0), the intrinsic *Q*_1*i*_ would have to be at least ~100 times larger than the loaded two-mode *Q*_2_ to have the same absolute dip-depth sensitivity:

(13) $$Q_{1i}=\left|\frac{1+x}{1-x}\right|\frac{2\sqrt{R_{+}}}{1-\sqrt{R_{+}}}Q_{2},$$

where *x* = *T*_1_/*αL*. Similarly, for the ideal one-mode case to have a linewidth sensitivity equal to the two-mode dip-depth sensitivity, the criterion is even stronger:

(14) $$Q_{1}=\frac{2\sqrt{R_{+}}}{1-\sqrt{R_{+}}}Q_{2},$$

meaning that the loaded one-mode *Q*_1_ needs to exceed the loaded two-mode *Q*_2_ by two orders of magnitude.

### 2.2. Experimental Setup and Procedure

As noted earlier, we detect the response (change in the dip depth or in the linewidth of a WGM) resulting from absorption by various concentrations of an analyte (SDC 2072 dye from H. W. Sands Corp., Jupiter, FL, USA) dissolved in methanol and interacting with the internal fraction *f* of a 1550 nm WGM in a hollow bottle resonator (HBR) [16,17]. Before using the microresonator, the absorption coefficients of the solvent and analyte were measured in a 2 mm cuvette. We found that *α_s_* = 8.76 cm^−1^, and that *α_a_* = 4.02 cm^−1^ at a concentration of 1 micromolar (µM), varying linearly with the concentration. Since this value of *α_s_* would not allow for the observed *Q* values on the order of 10^7^, the methanol absorption must be saturating in the microresonator. We checked this by focusing the laser beam through the cuvette, producing a peak intensity that we estimated to be less than that of the interacting fraction of the WGM. Under these conditions, we found *α_s_* = 2.2 × 10^–2^ cm^−1^, confirming the saturation; *α_a_* was unchanged.

**Figure 1:**
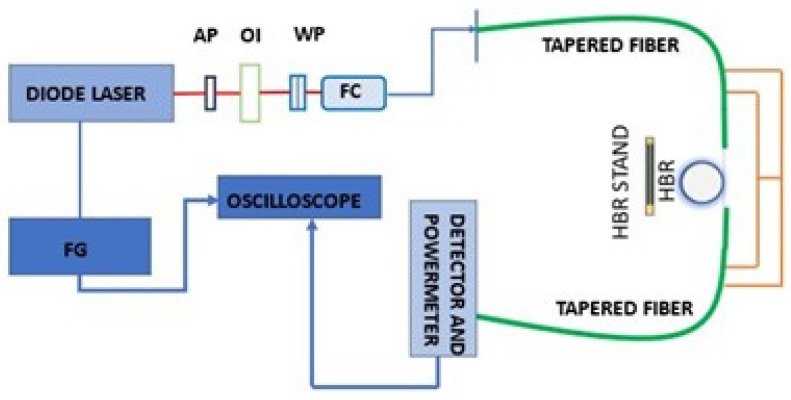
Illustration of the experimental setup.

An illustration of the experimental setup for dye absorption sensing is shown in Figure 1. A tunable diode laser spanning a wavelength range from 1508 nm to 1580 nm is used as the light source. A function generator FG is used to scan the laser in frequency. Before the light passes through a set of waveplates (WP), the beam passes through an anamorphic prism (AP) and an optical isolator (OI). The waveplates are used to select WGMs of one polarization. A fiber coupler (FC) is used to couple light into the tapered fiber, and a fiber isolator is used to prevent any back reflections arising from the tapered fiber. The light then travels through the tapered fiber and couples into and out of the microresonator. The signal is extracted at the other end of the tapered fiber and fed into a detector. The power meter receiving the detector signal is coupled to an oscilloscope that is triggered by the synchronization output of FG.

The tapered fiber in Figure 1 can be asymmetric, with a nonadiabatic downtaper and an adiabatic uptaper, for two-mode input, or symmetric, adiabatically bitapered, for one-mode input. The design, modeling, fabrication, and testing of the asymmetric fiber is described in detail in the next subsection. The symmetric fiber is fabricated to have the same waist radius as the asymmetric fiber.

**Figure 2:**
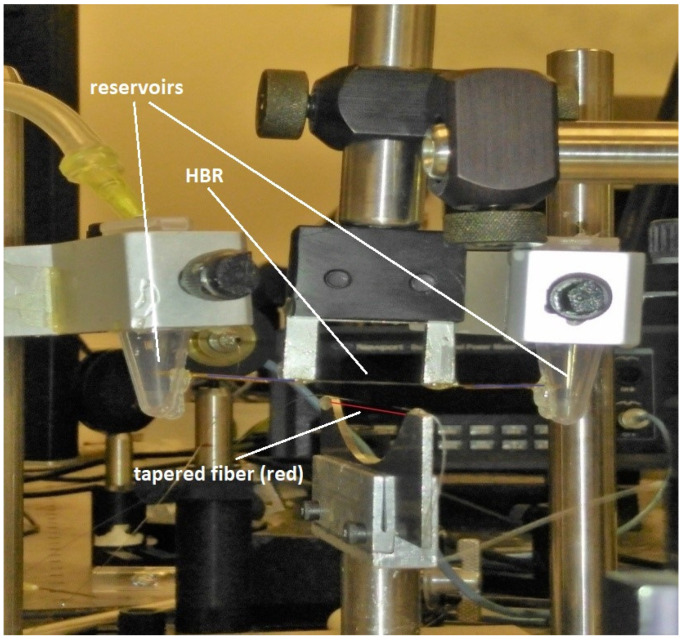
Closeup of the coupling region with the HBR microresonator and the tapered fiber (highlighted in red). In this photograph, the fiber and HBR have been separated for clarity. The reservoirs for the solvent and analyte are also shown.

Initially, an asymmetric tapered fiber was used to couple light into and out of a microresonator filled with methanol, and a WGM that showed a shallow throughput dip with the two input modes in phase and a small peak with the input modes out of phase was selected. Then, with the input modes in phase, an analyte at predetermined concentrations was added to the methanol, and changes in the dip depth and linewidth were recorded. Then, the asymmetric tapered fiber was replaced by a symmetric tapered fiber of the same waist radius, and, for the same WGM, changes in the dip depth and linewidth for different analyte concentrations were recorded for one-mode input. Tightly capped plastic vials were used as reservoirs for the analyte. The two ends of the microresonator were connected to the reservoirs, and the analyte inside one reservoir was pushed through the microresonator using a syringe feed at the top of that reservoir. A photograph of the part of the setup showing the tapered fiber, microresonator (HBR), and reservoirs is shown in Figure 2.

The ratio of fractional changes in the dip depth, asymmetric (two-mode) to symmetric (one-mode), for the same analyte concentration, gives the value of the enhancement *η*_21_, and the ratio of the fractional change in the dip depth (two-mode) to the fractional change in the linewidth (either case) gives the value of the enhancement *η_dl_*. The two-mode dip-depth data are $M(\alpha^{\prime }L)$ for various values of *α_a_*, which allows the value of the interacting fraction *f* to be determined. Note, however, that both enhancement factors are only weakly dependent on *f*, unlike the absolute sensitivity, which increases approximately linearly with *f*. The internal interacting fraction of a WGM will be, for absorption measurements, somewhat greater than the ratio of the power circulating in the inner region to the total power circulating in the mode [5]. In addition to having large internal interacting fractions, another advantage of the HBR is that it requires only a very small volume of the solvent and analyte [17].

### 2.3. Fabrication, Modeling, and Testing of Asymmetric Tapered Fiber

A tapered fiber is made by stretching a heated optical fiber, and it consists of a thin filament called the taper waist, each end of which is linked to the unstretched fiber by a section known as the taper transition. These taper transitions can be classified as adiabatic or nonadiabatic [18]. If the light propagating in the tapered fiber remains in the local fundamental mode (HE_11_) at all points along the taper transition, the transition is adiabatic; however, in a nonadiabatic taper transition, higher-order fiber modes are also excited, and hence, the light gets distributed among the local fundamental mode and higher-order modes as it travels along the tapered fiber.

**Figure 3:**
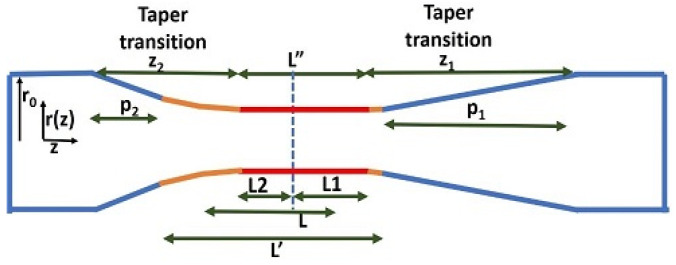
Asymmetric tapered fiber with nonadiabatic downtaper and adiabatic uptaper. Light propagates from left to right.

In our lab, the well-known “flame brush” technique is used to fabricate both symmetric and asymmetric tapered fibers. An optical fiber (SMF-28) with its jacket removed is attached to two motorized translation stages of a homemade fiber puller. Underneath the stripped area of the fiber, the flame of a hydrogen torch on another translation stage continuously brushes the stripped fiber along its length back and forth over a distance known as the brushing length *L*. While fabricating an asymmetric (nonadiabatic) tapered fiber, the two translation stages are pulled at different speeds, whereas a symmetric (adiabatic) tapered fiber is fabricated by pulling the two stages with the same speed. Much previous work has been conducted on the fabrication and characterization of asymmetric tapered fibers [19,20,21,22,23,24,25,26,27,28,29]. A schematic diagram of the asymmetric tapered fiber is shown in Figure 3, where light propagates from left to right. The downtaper transition (nonadiabatic) is relatively abrupt, whereas the uptaper transition (adiabatic) is more gradual. Thus, both fundamental and higher-order fiber modes will travel down the taper waist. However, the higher-order fiber modes will not survive the adiabatic uptaper and will be lost in the cladding.

To explain the labeling in Figure 3, first consider a symmetric tapered fiber. The fiber radius in the transition region is given by [22]

(15) $$r\left(z\right)=r_{0}\exp\left(-z/L^{\prime \prime }\right),$$

where *r*_0_ is the radius of the untapered fiber, *z* is the distance into the transition region, and $L^{\prime \prime }$ is the effective brushing length, i.e., the length of the taper waist. The waist radius is

(16) $$r_{w}=r\left(z_{0}\right)=r_{0}\exp\left(-z_{0}/L^{\prime \prime }\right),$$

where *z*_0_ is the transition length; in the symmetric limit of Figure 3, *z*_2_ = *z*_1_ = *z*_0_. As Figure 3 indicates, the waist length is not equal to the brushing length ($L^{\prime \prime }\neq L$), and the transition length is not equal to the pulling distance (*z*_0_ ≠ *p*_0_). The explanation for these effects is based on the following assumptions [30]: (i) the hydrogen flame has a finite width and, hence, the heated region $L^{\prime }$ extends 0.26 mm beyond the brushing length *L* on each end ($L^{\prime }=L+\;0.52\;\mathrm{mm}$); (ii) the end of the uniform waist is not at the end of the limit of the heated region, but is recessed inside it by a distance estimated to be *s* = 0.13 mm for the symmetric taper (so $L^{\prime \prime }=L^{\prime }-2s$), also implying that *z*_0_ = *p*_0_ + *s*. For a standard symmetric taper, *L* = 6.50 mm and *p*_0_ = 26.88 mm, so that

(17) $$r_{w}=(62.5\;\mu m)\exp\left(-27.01/6.76\right)=1.15\;\mu m.$$

For the asymmetric taper, we further assume [30] that the recession distance is inversely proportional to the pulling distance, or linearly dependent on the speed at which the other side of the taper is being pulled; this means that *s_i_* = *sp*_0_/*p_i_* (*i* = 1, 2), and so

(18) $$z_{i}=p_{i}+s_{i}=p_{i}+sp_{0}/p_{i}$$

and

(19) $$L^{\prime \prime }=L^{\prime }-s_{1}-s_{2}=L_{1}+L_{2}.$$

The two parts of the waist length have the same ratio as the lengths of the corresponding transition regions (*L*_1_/*L*_2_ = *z*_1_/*z*_2_), and so the waist radius is given by

(20) $$r_{w}=r_{0}\exp\left(-z_{i}/2L_{i}\right),$$

for either transition (*i* = 1 or 2). Support for this model is found when it is used in the fabrication of asymmetric and symmetric tapered fibers with the same waist radius, using them to couple light into the HBR. When the asymmetric fiber shows a shallow dip with the incident fiber modes in phase and a small peak with the incident modes out of phase, the symmetric fiber exhibits a resonance dip that goes nearly to zero throughput. Further confirmation through beat length measurements will be given below.

To see how adiabatic and nonadiabatic tapers can be differentiated, consider the local cladding taper angle Ω, defined as the angle between the fiber axis and the tangent to the taper profile at the point of interest:

(21) $$\Omega \left(z\right)=\left|\frac{dr\left(z\right)}{dz}\right|=\frac{1}{2L_{i}}r\left(z\right).$$

For a taper transition to be adiabatic, Ω must be less than some maximum value [20,31] at all values of the inverse taper ratio *r*(*z*)/*r*_0_. Strictly speaking, the condition to be satisfied is

(22) $$\Omega (z)=\left|\frac{dr\left(z\right)}{dz}\right|<<\left[k_{f}\left(z\right)-k_{h}\left(z\right)\right]r\left(z\right)$$

for all *z* in the taper transition, where *k_f_* and *k_h_* are the propagation constants of the fundamental and the higher-order fiber modes. These propagation constants can be calculated for the fiber (step-index dielectric waveguide) by solving the characteristic equation [32] at various taper radii in the taper transition.

As mentioned earlier, the relative phase of the fundamental and higher-order fiber modes varies with propagation, making the throughput profile of a nonadiabatic tapered-fiber-coupled microresonator system no longer a symmetric Lorentzian dip [33,34,35,36,37,38]. The distance between successive points where they are in phase is a beat length that can easily be measured by translating the fiber-HBR point of contact and finding the distance from one symmetric throughput dip (or peak) to the next. A practical implementation of Equation (22) is thus found by defining the maximum taper angle for the transition to be adiabatic:

(23) $$\Omega_{\max}(z)=\frac{\left[k_{f}\left(z\right)-k_{h}\left(z\right)\right]r\left(z\right)}{2\pi }=\frac{r(z)}{z_{b}},$$

where the beat length *z_b_* is given by:

(24) $$z_{b}=\frac{2\pi }{k_{f}\left(z\right)-k_{h}\left(z\right)}.$$

$\Omega_{\max}\left[r\left(z\right)/r_{0}\right]$ thus provides a delineation curve with which Ω(*z*) can be compared to determine the adiabaticity. Knowing the propagation constants at various taper radii allows us to calculate the beat length *z_b_*.

As light propagates down a taper transition, it makes a transition from core guidance (total internal reflection, TIR, at the core-cladding interface) to cladding guidance (TIR at the cladding-air interface). While it is core-guided, the light remains in the fundamental mode (HE_11_, also known as LP_01_); in this single-mode region, the propagation constant of the higher-order mode, *k_h_*, is taken to be the vacuum propagation constant times the cladding refractive index. Once the light becomes cladding-guided, we allow three possibilities for the higher-order mode, since the taper waist is multimode with a typical radius of 1.16 µm. These are: first, the LP_11_ family, consisting of TE_01_, TM_01_, and HE_21_; second, the LP_21_ and LP_02_ family, made up of HE_31_, EH_11_, and HE_12_, represented in the calculations by HE_12_; and third, the LP_31_ family (EH_21_ and HE_41_, represented by HE_41_).

**Figure 4:**
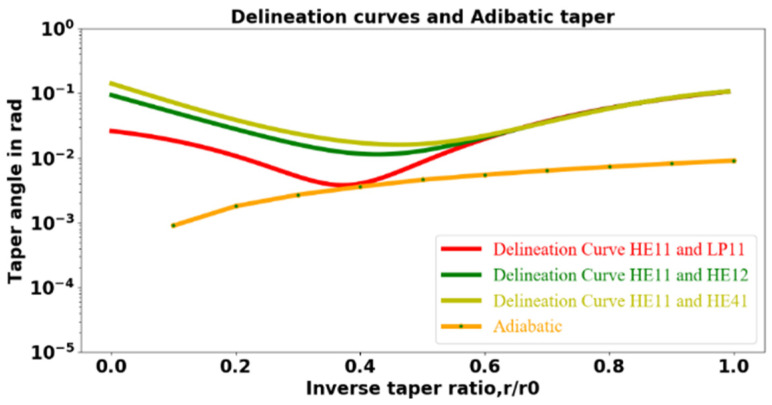
Delineation curves and plot of the cladding taper angle Ω as a function of the inverse taper ratio for an adiabatic taper to a waist radius of 1.15 μm.

Since we have three choices for *k_h_*, we will have three delineation curves. To plot each delineation curve, the larger of the Ω_max_ values for core guidance and cladding guidance at different values of the inverse taper ratio is used. These Ω_max_ values corresponding to different inverse taper ratios are fitted to polynomials and are shown in Figure 4, along with the Ω[*r*(*z*)/*r*_0_] for the adiabatic transition of a typical symmetric tapered fiber with a waist radius of 1.15 μm.

In Figure 4, the red, green, and yellow lines represent the different delineation curves. The red line is the delineation curve assuming the higher-order mode to be LP_11_ under the cladding guidance condition. In contrast, the green and yellow lines are the delineation curves assuming the higher-order mode to be HE_12_ and HE_41_, respectively. For all values of the inverse taper ratio, the orange line in Figure 4 representing the adiabatic taper will remain below the delineation curves, whereas for a non-adiabatic taper, the plot of the taper angle will pass above a delineation curve for at least some values of the inverse taper ratio. The correct delineation curve is determined by the experimental measurement of the beat length, as will be described below.

**Figure 5:**
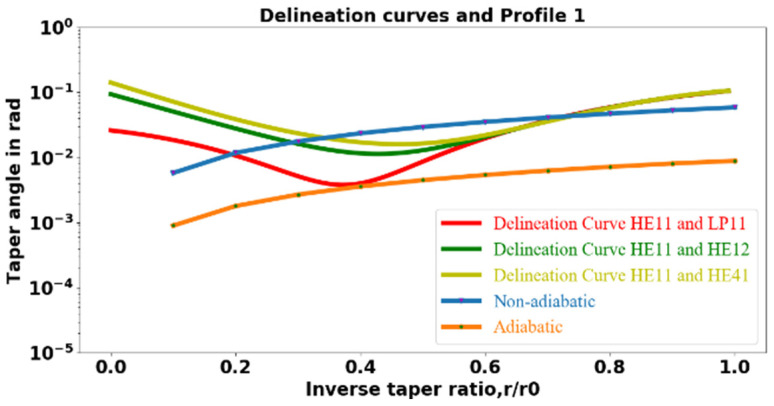
Delineation curves and plots of cladding taper angles Ω as a function of the inverse taper ratio for an asymmetric tapered fiber with a waist radius *r_w_* = 1.16 µm.

An asymmetric tapered fiber with one transition meant to be nonadiabatic and one meant to be adiabatic was fabricated, and the cladding taper angles are plotted along with delineation curves in Figure 5. In Figure 5, the red, green, and yellow lines represent the delineation curves. The blue curve represents the cladding taper angle for the abrupt downtaper transition. Since the blue curve passes above the minima of the delineation curves, the downtaper transition is nonadiabatic. The orange curve representing the taper angle for the uptaper transition passes below the delineation curves, indicating that the uptaper transition is adiabatic. Figure 5 thus implies that the fabricated asymmetric tapered fiber has a nonadiabatic downtaper and an adiabatic uptaper.

The average beat lengths of several asymmetric-tapered-fiber-coupled microresonator systems were measured using a screw-gauge actuated 3D stage to which the asymmetric fiber is mounted. The distance of translation of the fiber–HBR point of contact from where a symmetric throughput dip is observed to where the next symmetric dip is observed is the beat length. The experimentally measured beat length *z_b_* and the waist radius *r_w_* predicted by the model for three different asymmetric taper profiles are shown in Table 1. Profile 1 is the fiber whose taper transitions are plotted in Figure 5.

The experimentally measured beat lengths for the three different asymmetric taper profiles indicate that: (i) the modes responsible for beating are HE_11_ and LP_11_ and, hence, the delineation curve of interest is the red one in Figure 4 and Figure 5; and (ii) the radii predicted by the asymmetric taper fiber model for the three different taper profiles were very close to the radii estimated from the beat length measurements. It is worth noting that in addition to the HE_11_ and LP_11_ modes, other higher-order modes such as HE_12_ and HE_41_ may also be excited by light propagating along the nonadiabatic downtaper. Among all the higher-order modes, LP_11_ will be the most strongly excited, since the fiber sags during pulling [39]; this agrees with our beat length measurements shown in Table 1. Previously, it was shown that [6] by choosing a particular ratio of the resonator size to the diameter of the tapered fiber, only the HE_11_ and LP_11_ modes can significantly interact with the WGMs of a microresonator, and therefore, any weak excitation of other higher-order modes can be neglected. Thus, when different asymmetric taper profiles are used to couple light into the microresonator, only two modes, namely, HE_11_ and LP_11_, will significantly interact with WGMs.

## 3. Experimental Analysis and Results

The experimental procedure outlined in Section 2.2 was followed in several cases using each of the three asymmetric tapered fiber profiles of Table 1, accompanied by a symmetric fiber of the same waist radius. In all cases, the microresonator used was an HBR with an outer radius of (nominally) 90 µm and a wall thickness of ~14 µm, fabricated by heating and pressurizing a fused-silica capillary to form a bottle-shaped bulge. The small radius is what allows for significant coupling between the WGM and the higher-order fiber mode [6].

A detailed analysis of one such experiment using Profile 1 is presented below. The analysis consists of three parts. The first part (Section 3.1) compares the experimental two-mode vs. one-mode enhancement *η*_21_ to the theoretical prediction, whereas the second part (Section 3.2) compares the experimental and theoretical enhancements *η_dl_* for dip-depth vs. linewidth sensing. The third part (Section 3.3) compares the absolute sensitivity of the two-mode-input system to that of an *ideal* single-mode-input system.

### 3.1. Comparison of Experimental to Theoretical η_21_

**Figure 6:**
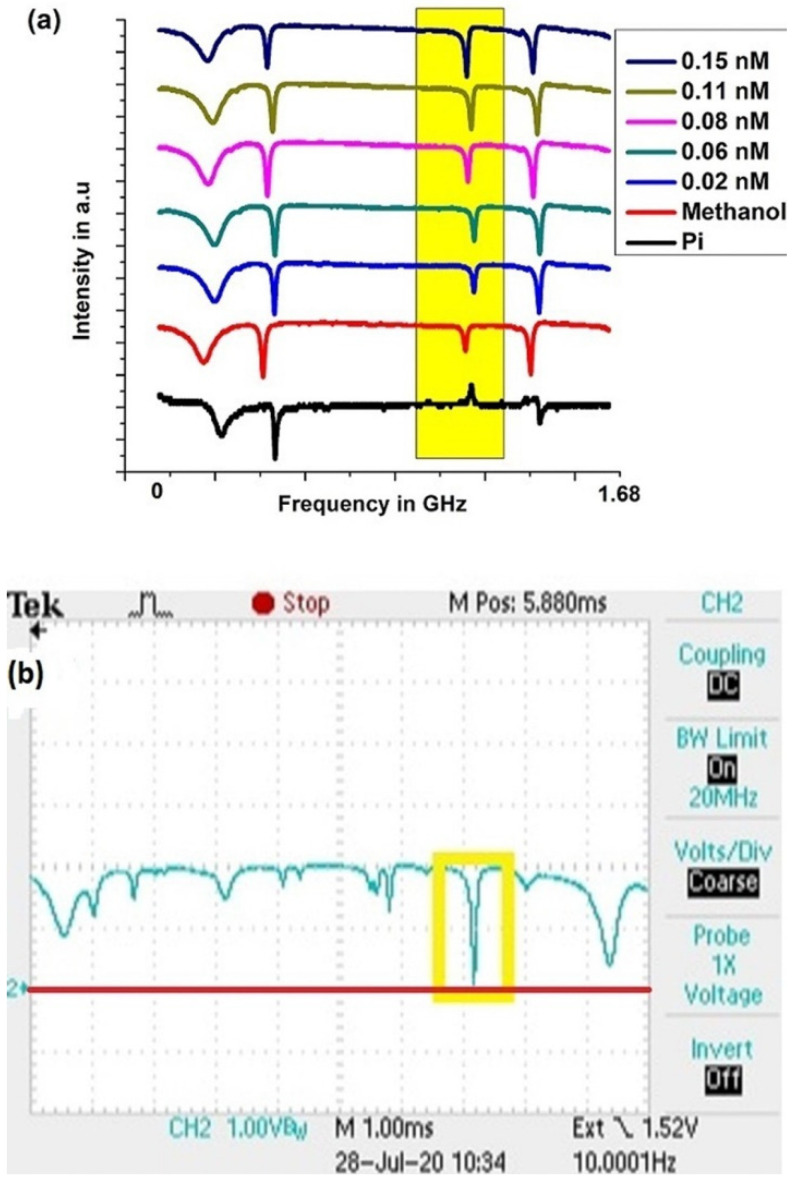
Throughput WGM spectra with input from tapered fibers of the same waist radius, *r_w_* = 1.16 µm. (**a**) Asymmetic tapered fiber. Upward displacement indicates an increasing analyte concentration (the order of traces is same as that in the legend); the bottom trace is for methanol only, with input modes out of phase. (**b**) Symmetric tapered fiber. Oscilloscope screenshot showing the mode of interest in the yellow box (the same mode as highlighted in (a); methanol only), very close to being critically coupled, as the bottom of the throughput dip is nearly at zero (the red line).

With an asymmetric tapered fiber used to provide two-mode input, WGMs that showed a shallow dip with the two input modes in phase, a small peak with the two modes *π* out of phase, and an increase in the dip depth with an increasing analyte concentration were the modes of interest for these sensing experiments. The throughput spectra of a tapered-fiber-coupled HBR system are shown in Figure 6, and a detailed analysis for the highlighted WGM is given below.

Initially, with only methanol inside the microresonator, measurements gave the following values: dip depth *M* = 0.035 ± 0.002, linewidth Δ*ν* = 13.52 ± 0.76 MHz, *R*_+_ = 0.965 ± 0.002, and *R*_−_ = 1.073 ± 0.002. From those measurements, the parameters in Equation (2) were calculated as shown in [15], and the enhancement in sensitivity predicted by the model was found using Equations (7) or (8) to be *η*_21_ = 2135 ± 132.

After adding analyte of predetermined concentrations, the corresponding dip depths *M*(*α’L*) were recorded and fitted to the model, with the interacting fraction *f* being the fitting parameter. (The resonant frequency of the WGM varies slightly for different analyte concentrations in Figure 6a because of small changes in temperature. Since we need to measure only the relative dip depth and the mode linewidth, this does not affect our results). By averaging the fitting parameters corresponding to various analyte concentrations, the interacting fraction *f* of the mode was found to be 0.062. Knowing *f* allows us to calculate the theoretical dip depth *M*(*α′L*) and the theoretical fractional changes in the dip depth Δ*M*/*M* and *dM*/*M*.

**Figure 7:**
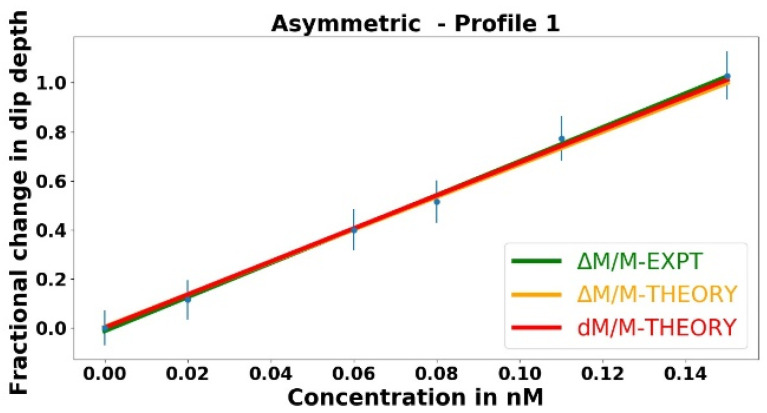
Fractional change in the dip depth plotted as a function of the analyte concentration, with the asymmetric tapered fiber used to couple light into and out of the microresonator.

With the asymmetric taper of waist radius 1.16 µm being used to couple light into and out of the resonator, the fractional change in the dip depth is plotted as a function of the analyte concentration in Figure 7. In Figure 7, the green curve represents the best fit to the experimental data points, whereas the orange and red curves represent the theoretical models. It is worth recalling that the dip depth *M* = 1 – *R*_+_ is found by measuring the throughput power (∝*R*_+_) collected on the detector. Note that |Δ *R*_+_| = |Δ*M*|, so even if Δ*M*/*M* is large, Δ*R*_+_/*R*_+_ will be small, and the experimental data Δ*M*/*M* will depend linearly on the concentration. The linear fit to the data points (green) was found using a Python program incorporating the experimental error shown by the error bars. The orange curve Δ*M*/*M*–*theory* represents the theoretical fractional change in the dip depth allowing for nonlinearity, and the red curve *dM*/*M*–*theory* represents the theoretical fractional change in the dip depth assuming linearity. At these low concentrations, the theoretical curves lie on top of each other, whereas this may no longer be true at higher concentrations. In Figure 7, our experiment agrees with the theory, well within the error limits.

**Figure 8:**
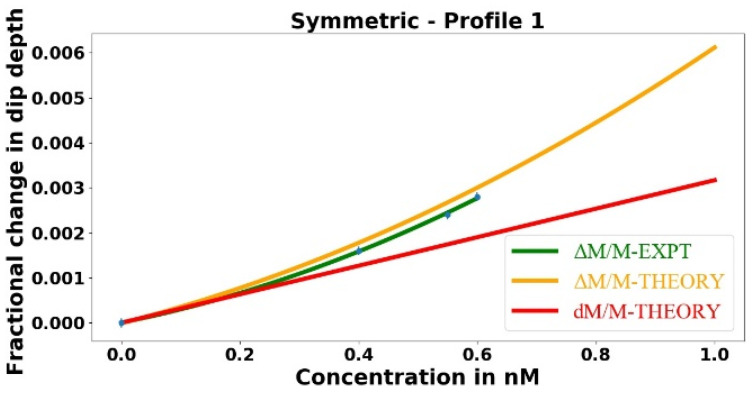
Fractional change in the dip depth plotted as a function of the analyte concentration, with the symmetric tapered fiber used to couple light into and out of the microresonator.

To experimentally demonstrate two-mode vs. one-mode enhancement *η*_21_, instead of the asymmetric tapered fiber a symmetric tapered fiber of approximately the same waist radius was used to couple light into and out of the HBR filled with methanol. In this case, the throughput spectrum for the WGM highlighted in Figure 6a shows near-critical coupling, as in Figure 6b. The linewidth of this WGM was measured and found to be Δ*ν* = 13.36 ± 0.15 MHz. This is equal (within experimental uncertainty) to the linewidth measured using the asymmetric fiber, indicating that the losses are the same in both cases, and confirming that both fiber waists have very nearly equal radii. The near-critical coupling necessitated the introduction of an offset voltage and scale expansion to follow changes in the bottom of the dip on the oscilloscope trace as analytes of various concentrations were added to the HBR. The fractional change in the dip depth is plotted as a function of the analyte concentration in Figure 8.

In Figure 8, the green curve represents the best fit to the experimental data points, whereas the theoretical curves are in orange and red. Since the mode of interest is close to critical coupling and has a dip depth *M* > 0.96, the relative throughput *R* < 0.04, and even if Δ*M*/*M* is very small, Δ*R*/*R* may be large, so quadratic fits to the experimental data and to Δ*M*/*M*–*theory* are performed. The red curve *dM*/*M*–*theory* represents the theoretical fractional change in the dip depth assuming linearity, and for very low concentrations, all three curves coincide. At higher concentrations, the experimental curve follows the trend of the Δ*M*/*M*–*theory* curve, indicating reasonable agreement.

Recall that the predicted enhancement factor is *η*_21_ = 2135 ± 132. At low concentrations, the experimental enhancement factor is calculated by taking the ratio of the slopes of the green curves shown in Figure 7 and Figure 8, giving *η*_21_ = 2616 ± 945. The uncertainties arise from system noise (mechanical, detector, and amplifier) on the measured throughput and are larger for the experimental value, which depends on the small *R* in the symmetric-fiber data, whereas the predicted enhancement has a smaller uncertainty from measuring the much larger values *R*_+_ and *R*_−_. Thus, the experimental enhancement agrees with the theoretical enhancement within the limits of uncertainty.

### 3.2. Comparison of Experimental to Theoretical η_dl_

This subsection contains the result of this work with the greatest practical significance: the sensitivity enhancement provided by two-mode dip-depth-change dissipative sensing relative to the standard linewidth-change sensing. The theoretical value of this enhancement is given by Equation (12), where the far right-hand side is approximately equal to 4/*M*, giving *η_dl_* = 114.3 ± 6.5. The fractional change in the dip depth is predicted to be approximately two orders of magnitude more sensitive than the fractional change in the linewidth.

**Figure 9:**
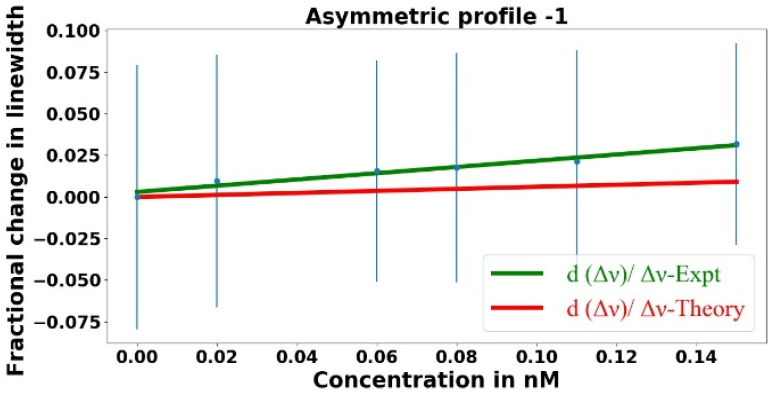
Fractional change in the linewidth plotted as a function of the analyte concentration, with the asymmetric tapered fiber used to couple light into and out of the microresonator.

Since the linewidth is linearly proportional to the total loss, as we increase the analyte concentration, the effective intrinsic loss *αL* increases, which in turn increases the total loss and the linewidth. Once again, knowing *f* and *α_a_* allows us to determine the theoretical linewidth using Equations (5) and (10). The fractional change in the linewidth is plotted as a function of the analyte concentration in Figure 9.

In Figure 9, the green line represents the best fit to the experimental data points, whereas the red line represents the theory. Since the linewidth is linearly proportional to the total loss, linear fits are performed to the experimental data points and theoretical values. The experiment and theory agree within the error limits, but there is huge uncertainty in measuring the very small change in the linewidth.

When the asymmetric tapered fiber is being used to find the fractional change in the linewidth, the experimental value of dip-depth vs. linewidth sensing enhancement is given by the ratio of the slopes of the green lines in Figure 7 and Figure 9. This gives *η_dl_* = 36.9 ± 109, which agrees with the theoretical prediction of 114.3 ± 6.5 within the huge error limit.

**Figure 10:**
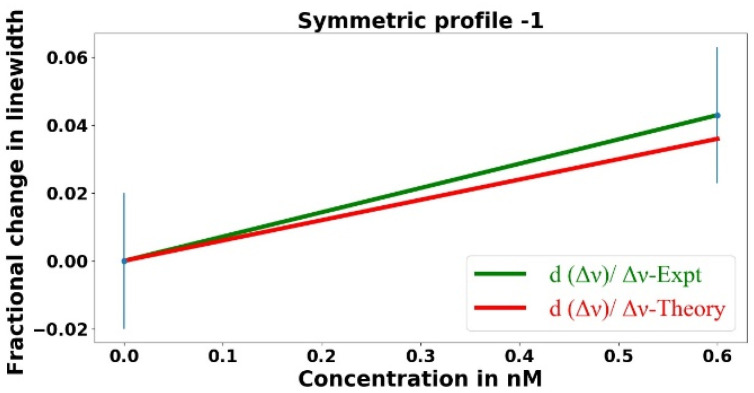
Fractional change in the linewidth plotted as a function of the analyte concentration, with the symmetric tapered fiber used to couple light into and out of the microresonator.

A better experimental value of this enhancement factor can be found by using the symmetric tapered fiber of the same waist radius, since the predicted change in the linewidth of the mode of interest will be the same for the same change in the analyte concentration. In this case, we measure the linewidth only twice, once with an analyte concentration of zero (only methanol inside the HBR) and again at the highest concentration of 0.6 nM, four times the maximum in Figure 9. The corresponding linewidths were found to be 13.36 ± 0.15 MHz and 13.93 ± 0.21 MHz, respectively, representing a precision about five times greater because of the greater dip depth. A plot of fractional change in the linewidth as a function of the analyte concentration is shown in Figure 10.

In Figure 10, the green line is the best fit to the experimental data points, whereas the red line represents the theoretical model. Now, taking the ratio of the slopes of the green lines in Figure 7 and Figure 10, we find *η_dl_* = 97 ± 46, which agrees better with the theoretical prediction of 114.3 ± 6.5. Because of the better precision in measuring the change in the linewidth, the experimental values of *η_dl_* will be calculated from the symmetric tapered fiber linewidth measurements.

### 3.3. Comparison of Absolute Sensitivity and Summary of Results

As discussed in Section 2.1, to achieve the same absolute sensitivity as that found with dip-depth sensing using two-mode input, but with *ideal* one-mode input, a greater WGM quality factor is needed. The ideal case is one in which the coupling is far from critical and there is negligible loss into higher-order fiber modes. The requirements on *Q*_1_ for the ideal system are given by Equations (13) and (14) for (one-mode) dip-depth and linewidth sensing, respectively. The best case obtains in the limits of strong overcoupling (*x* >> 1) or strong undercoupling (*x* << 1) when Equation (13) says that the intrinsic *Q*_1*i*_ of the ideal one-mode system must be approximately two orders of magnitude greater than the loaded *Q*_2_ of the two-mode system: (*Q*_1*i*_)_min_ = (4/*M*) *Q*_2_. For the case under consideration, since 4/*M* = 114.3 and *Q*_2_ = 1.43 × 10^7^, then (*Q*_1*i*_)_min_ = 1.64 × 10^9^. An intrinsic quality factor of the order of 10^9^ is near the limit of what can be achieved in fused silica without taking extraordinary measures. If the WGM excited in the ideal case is not strongly undercoupled or overcoupled, the value of *Q*_1*i*_ must be even greater.

A summary of a set of experiments (like the one described above) using the three asymmetric taper profiles of Table 1 is provided below in Table 2. In each case, the interacting fraction *f* was determined; the values are much larger than typical external evanescent fractions, showing one advantage of internal sensing in the HBR. Both experimental enhancement factors agree with their corresponding theoretical enhancement factors well within the error limits, confirming the theoretical analysis. In particular, the *η_dl_* enhancement factor shows that dip-depth dissipative sensing with two-mode input is approximately two orders of magnitude more sensitive than linewidth-based dissipative sensing. In comparing the absolute sensitivities, we find that an ideal one-mode system would require a *Q*_1*i*_ approximately equal to 10^9^ to have the same absolute sensitivity as our two-mode dip-depth sensing system with *Q*_2_ ~ 10^7^.

## 4. Discussion and Conclusions

The novel technique for enhancing microresonator-based dissipative sensing by using multimode input that we proposed earlier [15] is confirmed by the experimental work presented in this report. Multimode input produces an enhancement of approximately three orders of magnitude in the sensitivity of dissipative dip-depth-based sensing compared to the sensitivity achieved using single-mode input from a coupling fiber with the same waist radius. More importantly, this dip-depth-based sensing is enhanced by roughly two orders of magnitude over dissipative sensing based on the observation of changes in the WGM linewidth. The highlights of this technique are as follows. (i) The enhancements are independent of the quality factor *Q* of the WGM and independent of the value of the interacting fraction *f*. (ii) The enhancements do not depend on the input and output couplings being equal, and the method can be used at any wavelength in a microresonator of any type, size, or refractive index. (iii) Dip-depth sensing is advantageous because it is essentially independent of drifts or fluctuations in input power or WGM resonance frequency. (iv) The limit of detection for dip-depth sensing is enhanced by an additional factor of about 2.5 over linewidth sensing because the dip depth can be measured more precisely than the linewidth. (v) When an analyte absorption coefficient is known, this experimental method provides a means of determining the interacting fraction *f*. (vi) An absolute sensitivity comparable to that achievable by using a high-*Q* (~10^9^) WGM in an ideal (far from critical coupling) single-mode-input microresonator system can be achieved with multimode input on a resonator with a much more easily produced value of *Q* (~10^7^).

## Figures and Tables

**Table 1:** Taper profiles and beat length measurements.

Taper Profile	*p*_1_ (mm)	*p*_2_ (mm)	*L* (mm)	*r_w_* (µm)	*z_b_* (µm)
1	27.64	3.12	4.75	1.16	12.98
2	26.35	3.35	4.78	1.47	20.08
3	28.20	3.45	4.74	1.16	12.76

**Table 2:** Summary of results.

Taper Profile	*f*	Enhancement *η*_21_		Enhancement *η_dl_*		Comparison of Absolute Sensitivity	
		Theory	Expt	Theory	Expt	*Q*_2_	(*Q*_1*i*_)_min_
1	0.062	2135 ± 132	2616 ± 945	114.3 ± 6.5	97 ± 46	1.43 ×10^7^	1.64 × 10^9^
1	0.070	5548 ± 500	4286 ± 1467	149 ± 11	171 ± 71	1.29 × 10^7^	1.92 × 10^9^
1	0.052	1398 ± 62	2258 ± 931	45 ± 1	49 ± 12	1.62 × 10^7^	7.3 × 10^8^
2	0.110	1020 ± 38	956 ± 240	54.8 ± 1.5	43.1 ± 30.1	9.0 × 10^6^	4.9 × 10^8^
2	0.128	639 ± 19	735 ± 159	44.9 ± 1.0	43.4 ± 36.0	1.15 × 10^7^	5.2 × 10^8^
2	0.156	729 ± 23	886 ± 247	43.5 ± 0.9	45 ± 36	8.5 × 10^6^	3.7 × 10^8^
3	0.077	1166 ± 47	1094 ± 331	62.5 ± 2.0	76.6 ± 52.6	1.1 × 10^7^	6.96 × 10^8^
3	0.053	509 ± 33.4	545 ± 151	38 ± 1	37 ± 13	2.5 × 10^7^	9.5 × 10^8^

## Data Availability

Data are available from the corresponding author upon request.
